# Resveratrol inhibits Extranodal NK/T cell lymphoma through activation of DNA damage response pathway

**DOI:** 10.1186/s13046-017-0601-6

**Published:** 2017-09-26

**Authors:** Xianxian Sui, Canjing Zhang, Jianan Zhou, Shengxuan Cao, Chen Xu, Feng Tang, Xiuling Zhi, Bobin Chen, Songmei Wang, Lianhua Yin

**Affiliations:** 1 Department of Physiology & Pathophysiology, School of Basic Medical Sciences, Fudan University, Shanghai, China; 2 The Institution of Biomedical Sciences, Shanghai Medical College, Fudan University, Shanghai, China; 3 Department of Hematology, Huashan Hospital, Shanghai Medical College, Fudan University, Shanghai, China; 4 Shanghai Medical College, Fudan University, Shanghai, China; 5 Department of Pathology, Zhongshan Hospital, Shanghai Medical College, Fudan University, Shanghai, China; 6 Department of Pathology, Huashan Hospital, Shanghai Medical College, Fudan University, Shanghai, China; 7 Laboratory of Medical Molecular Biology, Experimental Teaching Center, School of Basic Medical Sciences, Fudan University, 131 Dongan Rd, Shanghai, 200032 China

**Keywords:** Extranodal NK/T cell lymphoma, Resveratrol, DNA damage response pathway, pATM, Epstein-Barr virus, Zta

## Abstract

**Background:**

Extranodal NK/T cell lymphoma (NKTCL) is a highly aggressive non-Hodgkin lymphoma with poor prognosis. Resveratrol (RSV, 3,5,4′-trihydroxystilbene), a natural nontoxic phenolic compound found in the skin of grapes and some other spermatophytes, performs multiple bioactivities, such as antioxidant activity, anti-aging activity, reduction of cardiovascular disease risk and anticarcinogenic effect. Here we report the anti-tumor effect of RSV in NKTCL cell lines SNT-8, SNK-10 and SNT-16.

**Results:**

RSV inhibited NKTCL cell proliferation in a dose- and time-dependent manner and arrested cell cycle at S phase. It induced NKTCL cells apoptosis through mitochondrial pathway, shown as down-regulation of MCl-1 and survivin, up-regulation of Bax and Bad, and activation of caspase-9 and caspase-3. In addition, we found that RSV suppressed the phosphorylation level of AKT and Stat3, and activated DNA damage response (DDR) pathway directly or through up-regulation of Zta of Epstein-Barr virus (EBV). Furthermore, using KU55933 as the inhibitor of pATM, we verified that DDR played an important role in RSV inducing NKTCL apoptosis. RSV also showed synergistic effect on activating DDR pathway in combination with etoposide or ionizing radiation, which resulted in cell proliferation inhibition and apoptosis.

**Conclusions:**

Our results provide in vitro evidence that RSV produces anti-tumor effect by activating DDR pathway in an ATM/Chk2/p53 dependent manner. So we suggest that RSV may be worthy for further study as an anti-tumor drug for NKTCL treatment.

**Electronic supplementary material:**

The online version of this article (10.1186/s13046-017-0601-6) contains supplementary material, which is available to authorized users.

## Background

Extranodal NK/T cell lymphoma (NKTCL) is a kind of rare but highly aggressive non-Hodgkin lymphoma which is commonly affecting Asians and Central and South Americans. The destructive lesions of nasal type NKTCL mainly occur in the nasal cavity, maxillary sinuses, or palate. And the lesions of extra-nasal type may involve any site, commonly skin, gut, and testes [1, 2]. NKTCL is characterized as clonal proliferation of natural killer cells or more rarely, T-cells. It is also known for close association with Epstein-Barr virus (EBV) which manifests a type II latent pattern [3]. EBV life cycle includes latent and lytic phase. During latent period, only a few genes are transcribed, such as LMP1, EBNA1, etc.. So the virus can escape the surveillance of hosts’ immune system [4, 5]. Once into lytic phase, more viral genes will be transcribed, such as Zta, BRLF1, BMRF1, etc., which result in viral replication [6]. Many studies suggest LMP1 is essential for EBV induced B-cell transformation in vitro. By constitutively deregulating p53, activating c-Myc and NF-κB pathway, LMP1 finally can up-regulate survivin expression and promote NKTCL cell survival [7]. A recent research indicates that LMP1 is a potential prognostic marker for patients treated with chemoradiotherapy [8]. The immediate early transactivator Zta (also referred to as BZLF1, ZEBRA, EB1), which can induce EBV into lytic phase and viral replication, has caused increasing interest in researchers [9]. Zta can cause genomic instability and render cells more sensitive to ionizing radiation or antiviral drugs [10]. Several tumor therapeutic strategies requiring the activation of EBV lytic genes for tumor cell killing have been described [11, 12].

For localized or early stage NKTCL disease, radiotherapy is the primary treatment. But treatment failure occurs in more than 50% of patients who are treated by radiation alone [13]. Besides, NKTCL tends to be resistant to conventional chemotherapy no matter localized or disseminated form. Some regimens such as SMILE (dexamethasone, methotrexate, ifosfamide, L-asparaginase, and etoposide), CHOP-L (L-asparaginase, cyclophosphamide, vincristine, doxorubicin and dexamethasone) have been devised to tackle these problems and result in better outcomes of patients in some extent [14, 15]. But the prognosis of NKTCL is still unsatisfying due to high incidence of toxicity and relapses despite many efforts of chemotherapy and radiotherapy have been made [1]. A prospective phase II study demonstrates that CHOP-L in combination with radiotherapy is promising for newly diagnosed NKTCL, but the strong myelotoxicity, liver dysfunction, and digestive tract toxicity are still disappointing [14]. Optimal treatment scheme comprising effective agents targeting NKTCL should be further explored [16]. Nowadays people realized that certain diet or diet components can control cancer development and progression. So the anti-tumor activities of some natural compounds have gained interest among researchers [17]. One of the natural compounds is resveratrol (RSV, 3,5,4′-trihydroxystilbene).

Resveratrol, a natural nontoxic phenolic compound found in the skin of grapes and some other spermatophytes, performs multiple bioactivities, such as antioxidant activity, anti-aging activity, insulin-sensitization, neuro-protection and reduction of cardiovascular disease risk [18]. Recently, more and more studies focus on the anticarcinogenic effect of RSV on many aspects (tumor cell growth, inflammation, apoptosis, angiogenesis, invasion and/or metastasis), as well as enhancing the sensitization of radiotherapies and chemotherapies, such as in bladder cancer and intestinal adenoma [19, 20]. The anti-tumor mechanisms of RSV have been preliminarily demonstrated, which include the activation of cell cycle arrest, cell apoptosis, DNA damage response (DDR) pathway, and modulating JAK2/Stat3 pathway, etc. [21, 22]. But the exact mechanism is still unclear.

Genomic damages, caused by various chemotherapeutic agents, ionizing, UV radiation or virus, can activate DDR, which results in DNA repair, chromatin remodeling, cell cycle arrest or cell apoptosis under different conditions [23, 24]. The kinase ataxia telangiectasia mutated (ATM) is a key mediator of DDR, which is induced by DNA double-strand breaks (DSBs). In DDR process, ATM is activated through autophosphotylation of serine 1981, which subsequently activates a range of downstream targets involved in cell cycle control, apoptotic response and DNA repair, such as checkpoint kinase 2 (Chk2), H2A.X, p53, cyclins, etc. [25]. DDR is an initial barrier to the emergence and development of some cancers. In the early stage of tumor, activated DDR can lead to cell senescence and apoptosis, resulting in delay or prevention of tumorigenesis [26, 27]. Previous researches showed RSV triggered DNA damage and directly activated ATM in some tumor cells [28, 29], but the effect of RSV in NKTCL is unknown.

In the present study, we described the biologic and molecular activities of RSV on NKTCL cells. Our results indicated that RSV induced EBV into lytic phase and activated DDR in NKTCL cells which resulted in cell apoptosis and cell cycle arrest. Our data suggests RSV may be a new therapeutic candidate for NKTCL.

## Methods

### Cell lines

The NKTCL cell lines SNT-8, SNK-10, SNT-16 (generous gifts from Dr. Norio Shimizu at Tokyo Medical and Dental University) were cultured in RPMI-1640 medium (Hyclone, Utah, USA) supplemented with 10% thermal inactivated human plasma and 700 U/ml of recombinant human interleukin-2 (IL-2) (Peprotech, NJ, USA). SNT-8 is an EBV positive NKTCL cell line derived from primary lesions of a Japanese patient. SNK-10 and SNT-16 are EBV positive NKTCL cell lines derived from the peripheral blood of patients with chronic active EBV infection [30].

### Cell proliferation assay

Cells were cultured in Phenol Red-free RPMI-1640 medium contained dimethyl sulfoxide (DMSO, as control), RSV (Sigma, louis, USA) or AG490 (Sigma, louis, USA). Cell viability was measured by Cell Counting Kit-8 (CCK-8, DOJINDO, Japan) following manufacturer’s protocol. The Cell viability ratio was calculated as below:

$$ \mathrm{Cell}\  \mathrm{viability}\  \mathrm{ratio}\ \left(\%\right)=\left[\mathrm{OD}\ \left(\mathrm{RSVor}\ \mathrm{AG}490\right)-\mathrm{OD}\ \left(\mathrm{Blank}\right)\left]/\right[\mathrm{OD}\ \left(\mathrm{Control}\right)-\mathrm{OD}\ \left(\mathrm{Blank}\right)\right]\times 100. $$

Each experiment was carried out in six replicates and results were calculated over three independent experiments.

### Cell cycle assay

Cells were incubated with DMSO or RSV for 24 h, cell cycle was measured using Cell cycle Detection Kit (KeyGEN, Nanjing, China) as manufacturer’s introduction. The DNA level was detected on FACSCalibur (BD, NJ, USA) using CELLQuest software (Becton Dickinson, Mountain View, CA).

### Cell apoptosis assay

Cells were cultured in 6-well plates with RPMI-1640 medium contained DMSO or different concentrations of RSV for 48 h. For ATM inhibition experiment, ATM inhibitor KU55933 (Selleck, Houston, USA) was added to culture medium 2 h before addition of 25 μM RSV. Ionizing radiation was performed at a dose of 4 Gy at room temperature after incubated with RSV for 2 h. The apoptosis effect of RSV was measured using Alexa Fluor® 488 annexin V−/Dead Cell Apoptosis kit (Life technology, Waltham, USA) following manufacturer’s protocol. Cell apoptosis was performed on FACSCalibur (BD, NJ, USA) using CELLQuest software (Becton Dickinson, Mountain View, CA). The data were analyzed using Flowjo software.

### Realtime PCR

Total RNA was extracted using TRIzol Reagent (life technologies, Carlsbad, USA) following manufacturer’s protocol. cDNA was synthesized using ReverTra Ace qPCR RT Kit (Toyobo, Osaka, Japan). Realtime PCR was performed with SYBR® Green qPCR Master Mix (Bio-Rad, Hercules, USA) on a CFX96 Touch™ Real-Time PCR Detection System. β-actin was detected as a loading control. Primers were as follows:

β-actin-Forword: 5′-ACTGGAACGGTGAAGGTGACAG-3′, β-actin-Reverse: 5′-GGTGGCTTTTAGGATGGCAAG-3′; BZLF-1-Forword: 5′-TACAAGAATCGGGTGGCTTC-3′, BZLF-1-Reverse: 5′-GCACATCTGCTTCAACAGGA-3′; LMP-1-Forword: 5′-CCCTTTGTATACTCCTACTGATGATCAC-3′, LMP-1-Reverse: 5′-ACCCGAAGATGAACAGCACAAT-3′. Relative expression of RNA was determined as below:

The relative expression = 2 ^-(ΔΔCT)^, where ΔCT = (cycle threshold (CT) detected gene) - (CT β-actin) and ΔΔCT = ΔCT (RSV) - ΔCT (Control).

### Western blot analysis

Cells were lysed in RIPA Lysis Buffer (Beyotime, Nantong, China) containing phosphatase inhibitor (Roche, Basel, Switzerland) and PMSF (Beyotime, Nantong, China). Total proteins were quantitated by using Enhanced BCA Protein Assay Kit (Beyotime, Nantong, China). Protein samples were separated by SDS-PAGE and transferred to PVDF membranes (Immobilon-P membrane, Millipore, USA). The membranes were blocked with 5% non-fat milk for 1 h at room temperature in TBST, and then probed with primary antibodies overnight at 4 °C. After washed three times in TBST, the membranes were incubated with secondary antibodies for 1.5 h at room temperature. Membranes were washed another three times in TBST and then detected by using WesternBright™ ECL (ComWin Biotech, Beijing, China) on Tanon 5200 (Tanon, Shanghai, China).

#### Antibodies

Anti-phospho-AKT (Ser473), anti-AKT, anti-phospho-JAK2 (Tyr1007/Tyr1008), anti-JAK2, anti-phospho-Stat3 (Tyr705), anti-Stat3, anti-phospho-Chk2 (Thr68) and anti-phospho-p53 (Ser15) antibodies were purchased from Cell Signaling Technology (Boston, USA). Anti-Bax, anti-Bcl-2, anti-Mcl-1, anti-Caspase-3, anti-Caspase-9, anti-Survivin, anti-Cyclin A2, anti-phospho-ATM (Ser1981), anti-ATM, anti-γH2A.X (Ser139) and anti-LMP1 antibodies were purchased from Abcam (Cambridge, UK). Anti-pBad (S136) and anti-Bad were purchased from Bioworld Technology (Minneapolis, USA). Anti-p53 and anti-Zta antibodies were purchased from Santa Cruz Biotechnology (Texas, USA). Anti-β-actin and HRP-conjugated goat anti-mouse/rabbit secondary antibodies were purchased from ComWin Biotech (Beijing, China).

### Immunofluorescence analysis of the phosphorylation level of ATM and Zta

SNT-8 cells were cultured in the presence of DMSO or RSV for 6 h. Cells were washed two times in PBS, fixed in 4% paraformaldehyde for 30 min, and then pipetted onto glass slides pre-treatment with polylysine. Cells were permeabilized in 0.2% Triton X-100 for 10 min and blocked in 3% BSA. Then incubated with Anti-Phospho-ATM (Ser1981) and anti-Zta primary antibody overnight at 4 °C. The slides were washed in PBS and then incubated with goat anti-rabbit CY3 or goat anti-mouse Alexa Fluor 488 (Goodbio, Wuhan, China) antibodies and for 1 h at room temperature and further stained with DAPI. Confocal images were acquired by NIKON C2 confocal laser-scanning microscope (Nikon, Tokyo, Japan).

### Statistical analysis

Data were presented as mean ± SD and analyzed by *one-way ANOVA* using SPSS19.0*.* A value of *p* < 0.05 was considered statistically significant. The results were performed with Graphpad software (Graphpad software, San Diego, CA).

## Results

### RSV inhibits the proliferation of NKTCL cells

**Fig. 1:**
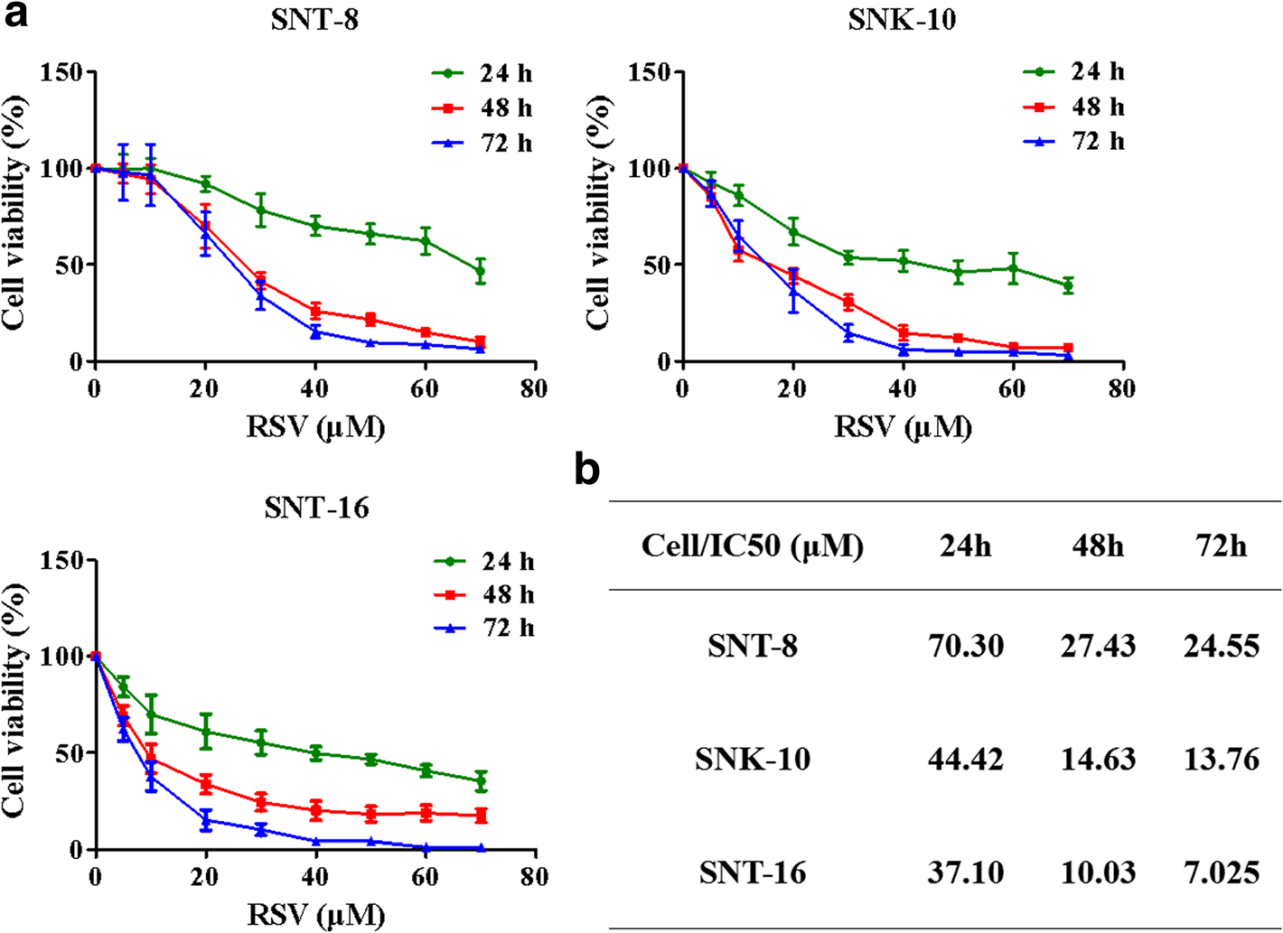
RSV inhibits the proliferation of NKTCL cells. SNT-8, SNK-10 and SNT-16 cells were treated with RSV in the concentration of 0 μM (Control), 5 μM, 10 μM, 20 μM, 30 μM, 40 μM, 50 μM, 60 μM, or 70 μM for 24 h, 48 h or 72 h respectively. **a** The cell proliferation viability effect of RSV was measured by CCK-8 assay (*n* = 3). **b** IC_50_ of RSV on SNT-8, SNK-10 and SNT-16 cells

We investigated the effects of RSV on NKTCL cell viability using the CCK-8 assay. SNT-8, SNK-10, and SNT-16 cells were treated with different concentration of RSV for different time. We found that RSV significantly inhibited the proliferation of three NKTCL cell lines in a dose- and time-dependent manner (Fig. 1a). The IC_50_ at different time was shown in Fig. 1b.

### RSV arrests NKTCL cell cycle at S phase

**Fig. 2:**
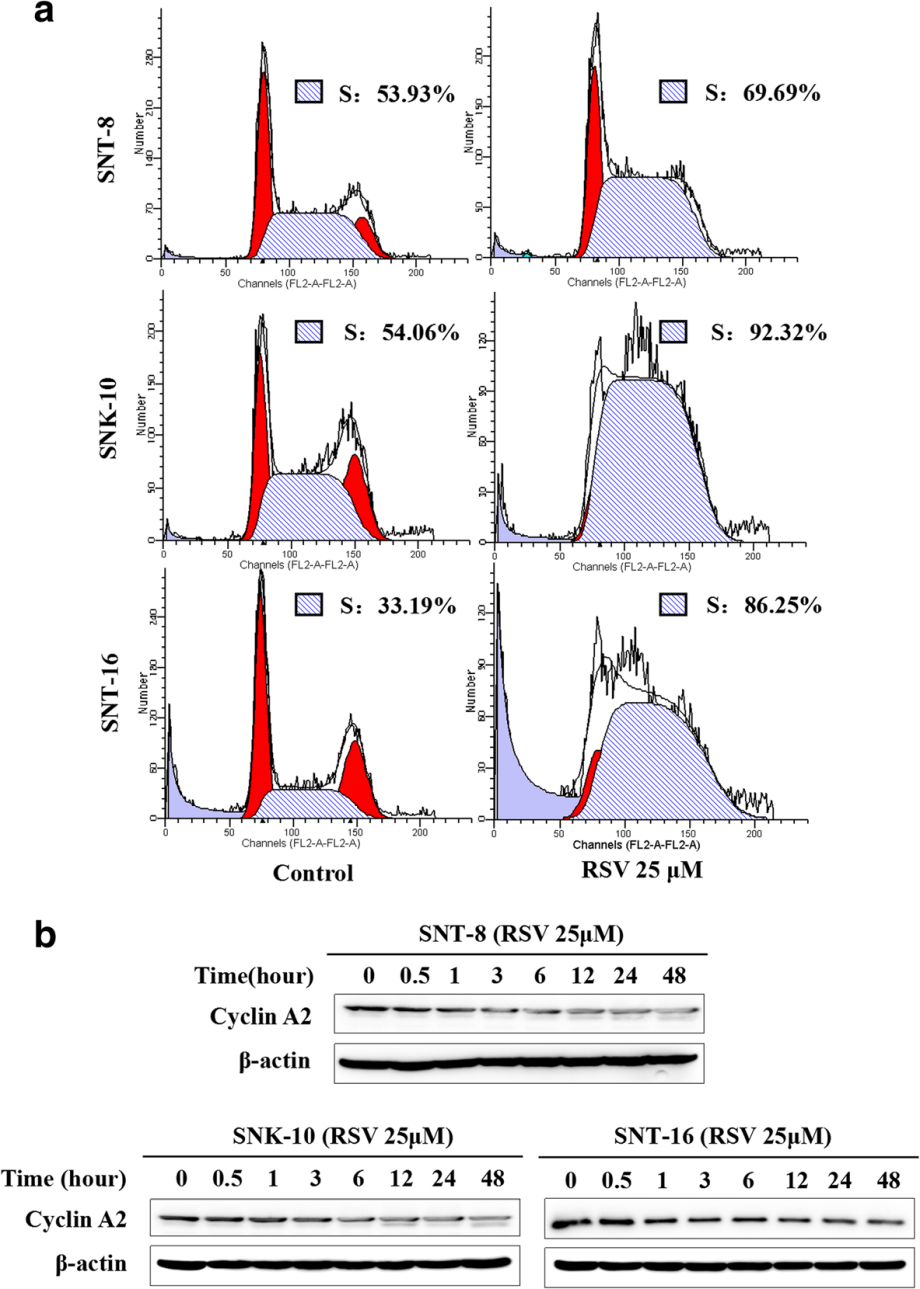
RSV arrests NKTCL cell cycle at S phase. **a** Cells were treated with 25 μM RSV for 24 h. After PI staining, the DNA content was measured by Flow cytometry (n = 3, S phase was marked in forward slash). **b** The expression of Cyclin A2 in cells was detected by western blot analysis after treated with RSV for different time. β-actin was used as a loading control

Cell cycle analysis was performed by Flow cytometry using PI staining. The results showed that RSV significantly increased the percentage of S phase cells, while decreased the percentage of G1 and G2/M phase cells (Fig. 2a). Further, we checked the expression of Cyclin A2, a protein which is essential for the control of cell cycle at the S and G2/M phase transition. As shown in Fig. 2b, RSV inhibited the expression of Cyclin A2.

### RSV induces NKTCL cells apoptosis through mitochondria-mediated caspase pathway

**Fig. 3:**
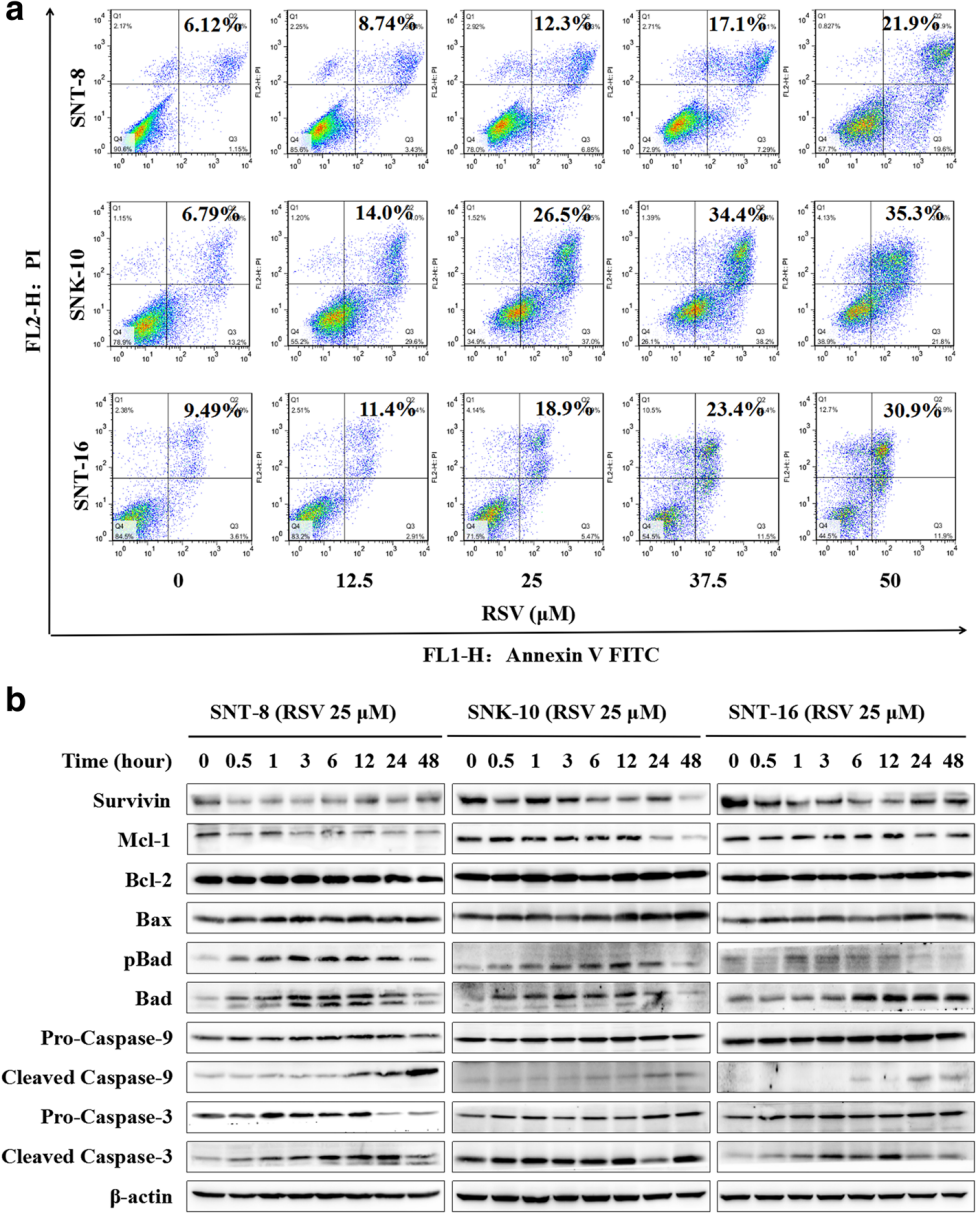
RSV induces NKTCL cells apoptosis through mitochondria-mediated caspase pathway. **a** We used the Annexin V-FITC apoptosis detection kit to determine cell death level. SNT-8, SNK-10 and SNT-16 cells were treated with RSV in different concentration for 48 h. Cell apoptosis rate was evaluated by Flow cytometry. **b** Western blot analysis of the expression of Survivin, Mcl-1, Bcl-2, Bax, Bad, caspase-9, cleaved-caspase-9, caspase-3 and cleaved-caspase-3 in cells treated with RSV for different time. β-actin was used as a loading control

We investigated the effects of RSV on NKTCL cell apoptosis using FITC-conjugated Annexin V and PI staining. As shown in the flow cytometry histograms, RSV increased apoptosis percentage of NKTCL cells in a dose-dependent manner (Fig. 3a). To determine which pathway participated in the apoptosis of resveratrol, Survivin, Bcl-2 and caspase families were detected using western blot analysis. The data showed that RSV had no obvious effect on the expression of Bcl-2 while it down-regulated Mcl-1 and survivin, and up-regulated Bax and Bad. Furthermore, it increased the expression of cleaved caspase-9 and cleaved caspase-3 (Fig. 3b). These results suggest that RSV induces apoptosis through mitochondria-mediated caspase pathway in NKTCL cell lines.

### RSV inhibits cell proliferation through decreasing phosphorylation level of AKT and Stat3 in NKTCL cells

**Fig. 4:**
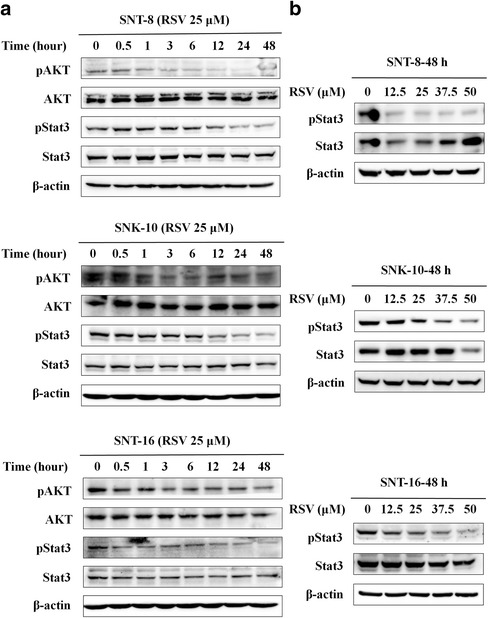
RSV inhibits phosphorylation level of AKT and Stat3. Expression level of AKT, pAKT, Stat3 and pStat3 in cells was determined by western blot analysis. **a** Cells were treated with 25 μM RSV for different hours. **b** Cells were treated with RSV in different concentration at 48 h

In NKTCL, AKT and JAK/Stat3 pathways were often over activated. In our study, we found RSV decreased the phosphorylation level of AKT and Stat3 at different time in all of the three cell lines (Fig. 4). In order to demonstrate whether Stat3 phosphorylation is directly associated with cell proliferation in NKTCL cells, we inhibited Stat3 by using AG490, a JAK2 inhibitor. We found that AG490 decreased the phosphorylation level of JAK2 and Stat3 (Additional file 1: Figure S1B), while inhibited the proliferation of SNT-8 and SNK-10 cells in a dose-dependent manner (Additional file 1: Figure S1A). That means RSV can inhibit AKT and JAK/Stat3 pathway in NKTCL cells through inhibiting the phosphorylation level of AKT and Stat3.

### RSV shows anti-tumor activities though activating DNA damage response pathway in NKTCL cells

**Fig. 5:**
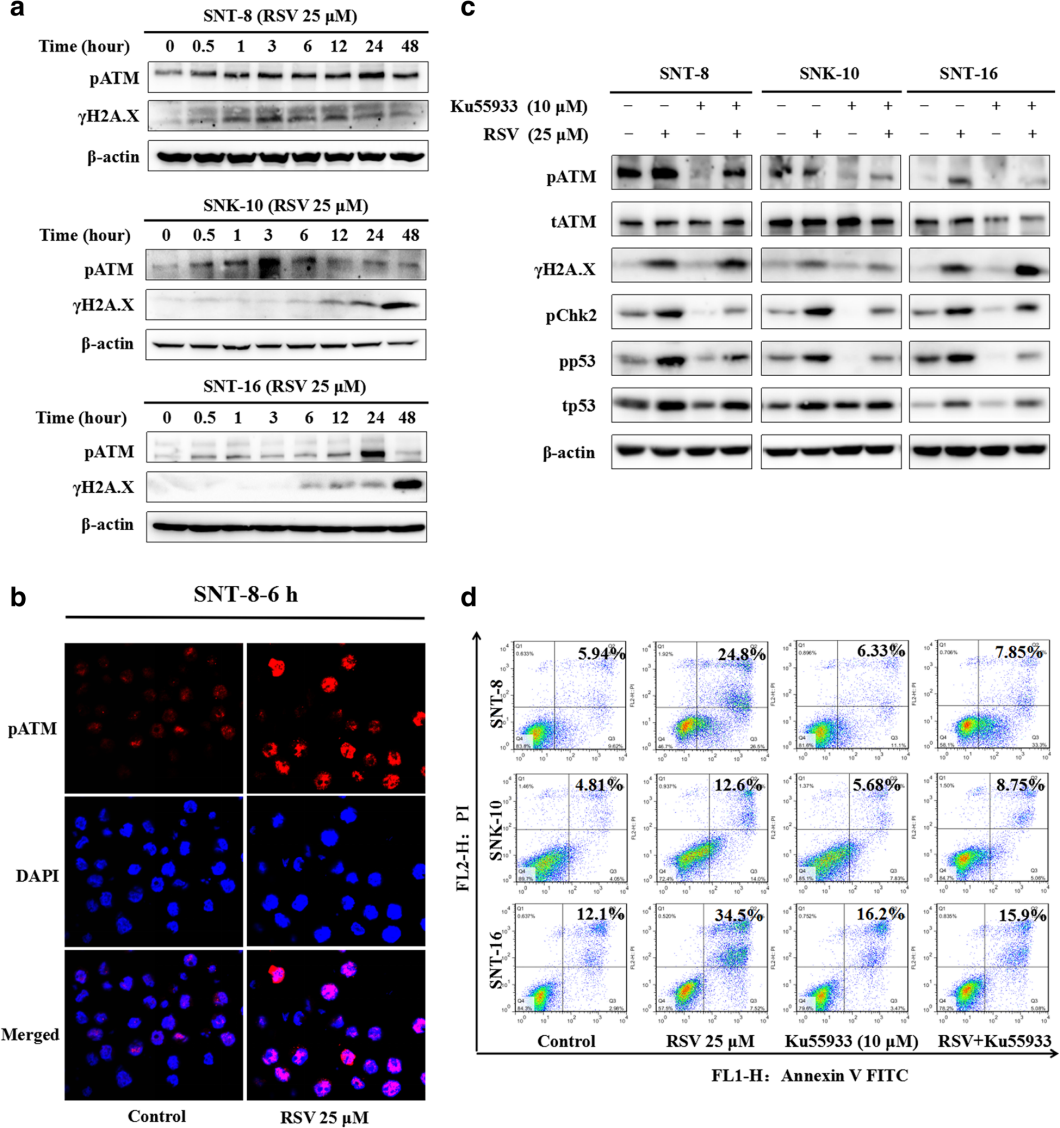
RSV actives DNA damage response pathway in NKTCL cells. **a** Cells were treated with 25 μM RSV for different time, the protein level of pATM (S1981) and γH2A.X (S139) in SNT-8, SNK-10 and SNT-16 cells was detected with western blot analysis. **b** The expression and foci of pATM (S1981) in SNT-8 cells after treated with 25 μM RSV for 6 h was determined by immunofluorescence. Cell nuclei were stained by DAPI. Images were acquired using confocal laser-scanning microscope (Scale bar, 20 μm). **c** Cells were incubated with RSV (25 μM), ATM inhibitor KU55933 (10 μM), or the combination of RSV and KU55933 for 6 h. The protein levels of pATM (S1981), ATM, γH2A.X (S139), pChk2 (T68), pp53 (S15), p53 were monitored. RSV enhanced the expression of pATM, and its down-stream molecules pChk2, γH2A.X and pp53 as well. While KU55933 inhibited the phosphorylation of ATM, p53, Chk2, but not H2A.X. When combined with RSV, KU55933 can diminish the activation level of pATM, pChk2 and pp53 induced by RSV, but not γH2A.X. **d** Cells were incubated with DMSO, RSV (25 μM), ATM inhibitor KU55933 (10 μM), or the combination of RSV and KU55933 for 48 h. The apoptosis was analyzed by Flow cytometry in each group. RSV alone increased cell apoptosis, while KU55933 alone showed no effect. When combining RSV and KU55933 together, the increased apoptosis induced by RSV was reversed

To observe the influence of RSV on DDR pathway in NKTCL cells, we examined the change of two key molecules during DDR: pATM and γH2A.X. As shown in Fig. 5a, the expression of pATM and γH2A.X significantly increased in NKTCL cells after being treated with RSV for different time. We next detected the expression of pATM in control and RSV treated cells by immunofluorescence and images were captured using confocal microscope. It demonstrated that RSV increased the expression of pATM in SNT-8 cells (Fig. 5b).

Furthermore, to verify whether ATM is essential during RSV inducing NKTCL cell apoptosis, we investigated the change of cell apoptosis induced by RSV with or without the ATM inhibitor KU55933 in the three cell lines. As shown in Fig. 5c, RSV increased phosphorylation level of ATM while had no influence on ATM expression level. Meanwhile the phosporylation level of ATM’s downstream molecules H2A.X, Chk2 and p53 were also raised (Fig. 5c). The apoptotic proportions of cells were also increased after being treated with RSV for 48 h (Fig. 5d). As an ATM inhibitor, KU55933 alone reduced the phosphorylation of ATM, Chk2 and p53 in all three cell lines, but had no influence on cell apoptosis in any NKTCL cell lines (Fig. 5c and d). Combined with KU55933, RSV still increased γH2A.X, but only caused very little change on the expression of pATM, pChk2 and pp53, compared with those in control cells. Notably, in the presence of KU55933, RSV induced apoptosis was reverted to control level in all the three cell lines. These results suggest that DDR pathway especially pATM is essential in RSV inducing apoptosis of NKTCL cells.

Etoposide, which can cause DNA strands to break and promote cell apoptosis, is one of the important anti-cancer drugs in NKTCL treatment in clinical setting. To further investigate and characterize the effect of RSV alone or in combination with etoposide on DDR, we examined the changes of pATM, γH2A.X, pChk2 and p53. As shown in Additional file 2: Figure S2B, the combination treatment enhanced the expression of these proteins in contrast to RSV or etoposide alone. Beside, RSV in combination with etoposide has synergistic effect on the inhibition of NKTCL cells proliferation (Additional file 2: Figure S2A).

In addition, as ionizing radiation also induces DDR, and radiotherapy combined with chemotherapy is a common treatment strategy for NKTCL, the effect of RSV combination with ionizing radiation for treatment of NKTCL was examined. The results showed that ionizing radiation induced cell apoptosis, and the effect was strengthened when in combination with RSV (Additional file 3: Figure S3A). Combination with ionizing radiation also has synergistic effect on DDR activation (Additional file 3: Figure S3B).

Taken together, these results suggest that RSV showed anti-tumor activities through activation DDR pathway in NKTCL cells.

### RSV induces EBV into lytic phase in NKTCL cells

**Fig. 6:**
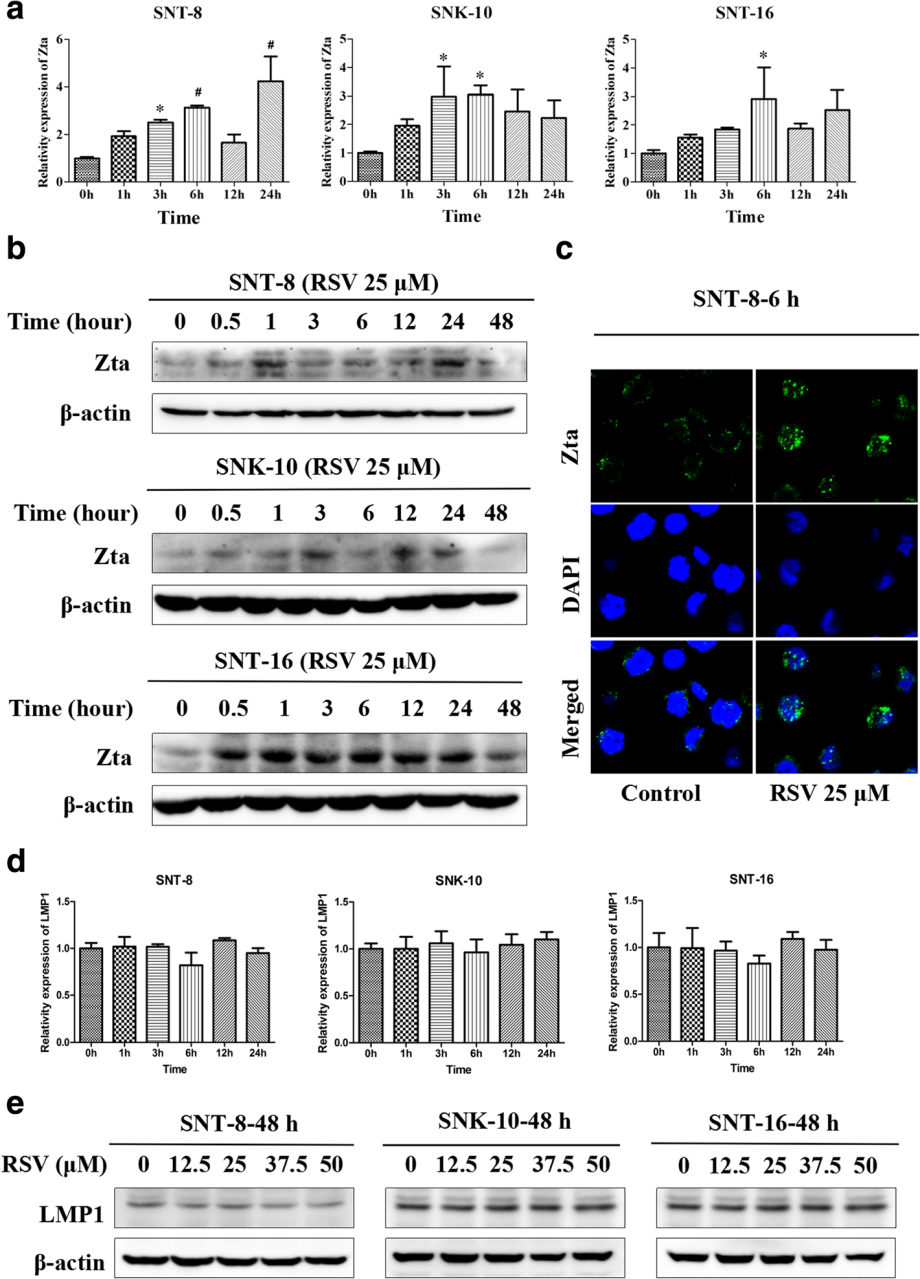
RSV induces EBV into lytic phase in NKTCL cells and has no effect on the expression of LMP1 in mRNA and protein levels. **a** The mRNA level of EBV lytic gene Zta in cells after treated with RSV for different hours was measured using Real-time PCR assay (n = 3).*p < 0.05, ^#^ *p* < 0.01 vs control. **b** Protein level of Zta in cells was determined by western blot analysis. Cells were treated with 25 μM RSV for different hours (n = 3). **c** Protein level of Zta was detected by immunofluorescence in cells incubated with RSV for 6 h. Cell nuclei were stained by DAPI. Images were acquired using confocal laser-scanning microscope (Scale bar, 20 μm). **d** The mRNA level of LMP1 in cells after treated with RSV for different hours (n = 3).*p < 0.05, ^#^ *p* < 0.01 vs control. **e** Protein level of LMP1 in cells after treated with RSV in different concentration at 48 h

Since NKTCL was highly related to EBV, we examined the expression of lytic gene Zta and latent gene LMP1 of EBV in NKTCL cells after incubating with RSV. The results showed that RSV significantly increased the expression of Zta on both mRNA (Fig. 6a) and protein levels (Fig. 6b) in all of the three cell lines. Furthermore, we examined the expression of Zta in SNT-8 cells by immunofluorescence. As shown in Fig. 6c, Zta was increased in RSV treated cells. However, there was no obvious influence of RSV on LMP1 expression either on mRNA (Fig. 6d) or protein level (Fig. 6e).

## Discussion

A great variety of researches have reported the anti-proliferation and pro-apoptotic activity of RSV against various human cancer cell lines and animal models since 1997 [31]. And the multiple mechanisms have been investigated at molecular, cellular and physiological levels [32]. RSV is not toxic to human peripheral blood mononuclear cells (PBMCs) while is cytotoxic to lymphoma and leukemia cells at the same concentration [33–36]. Clinical trials demonstrate that RSV does not cause any adverse events in healthy volunteers or in chronic lymphocytic leukemia and colon cancer patients [37–39]. However, the effect of RSV on NKTCL is unknown. Our results demonstrated RSV inhibited the proliferation of SNT-8, SNK-10 and SNT-16 cells with IC_50_ ranged from 7 μM to 70 μM at different time. It also induced NKTCL cell cycle arrest and apoptosis. Therefore, RSV could inhibit the growth of NKTCL cells by at least partially inducing apoptosis and cell cycle arrest.

The Bcl-2 families (Mcl-1/Bcl-2/Bax/Bad) and caspase protein families play important roles in mitocnondrial (intrinsic) pathway of apoptosis. Increased pro-apoptotic protein Bax/Bad and decreased anti-apoptotic protein Mcl-1/Bcl-2 can stimulate the release of cytochrome c from mitochondria, which activates initiator caspase, caspase-9. Caspase-9 then catalyzes the activation of executioner caspase, caspase-3, ultimately leading to apoptosis [40]. Survivin, a member of the inhibitor of apoptosis (IAP) family, functions to inhibit caspase activation, thereby leading to negative regulation of apoptosis [41]. Previous data have shown that RSV decrease the expression of Mcl-1 in T-cell acute lymphoblasticleukemia cells [42], up-regulated Bax in Burkitt’s lymphoma cells [34], activated caspase-9 in acute lymphoblastic leukemia cells [36] and induced the cleavage of caspase-3 in human T-cell leukemia virus type 1 (HTLV-1) [43]. In our study, RSV suppressed the expression of Mcl-1 and survivin, up-regulated Bax/Bad, activated caspase-9 and caspase-3, but it had little influence on Bcl-2. These data suggest that RSV can induce NKTCL cell apoptosis through mitochondrial pathway.

In NKTCL, AKT and JAK/Stat3 pathways are constitutively activated and promote cell survival in large part by inhibiting apoptosis. Studies have demonstrated that activated AKT could directly phosphorylate and inhibit the function of pro-apoptotic Bcl-2 family members and then offer growth advantages [44–47]. Our results showed a down regulation of pAKT and pStat3 in RSV treated cells. Incubating with AG490, a JAK2 inhibitor, the phosphorylation level of JAK2 and stat3 was suppressed in SNT-8 and SNK-10 cells. Meanwhile, cell proliferation inhibition was observed. These results suggests that Stat3 phosphorylation is directly associated with NKTCL cell proliferation, and the decreased pAKT and pStat3 may play a promoting role in RSV-induced NKTCL cell growth arrest and apoptosis.

Present study indicates that DDR is commonly activated in majority of tumors including urinary bladder, breast, lung and colon [27]. Notably, RSV induced ATM activation is accompanied by S phase arrest in ovarian tumor cells, malignant B cells and leukemia cells [48–50]. Our data reinforced this point by showing that RSV can inhibit NKTCL cell growth and induce cell arrest at S phase and apoptosis by at least partially enhancing DDR pathway.

DSBs induce the activation of ATM, and then ATM phosphorylates Chk2 on threonine 68, which subsequently phosphorylates a range of proteins involved in cell cycle control and apoptosis, such as p53 and BRCA1 [51]. p53 can up-regulate cell cycle inhibitors such as the CDK inhibitor p21, leading to the down-regulation of cyclins and cell cycle arrest [52, 53]. Also, p53 can induce cell apoptosis though Bcl-2 family and finally activate caspase cascade [54]. In NKTCL cell lines, we found RSV activated DDR though up-regulation of pATM and γH2A.X, which are early cellular response molecules to the generation of DSBs [55]. Furthermore, the phosphorylation level of ATM’s downstream molecules Chk2 and p53 was also increased. We suppose that p53 inhibits Cyclin A2 through p21, which ultimately leading to the cell arrested at S phase. Also, p53 could activate Bax/Bad and caspase cascade, which leading to cell apoptosis. To confirm DDR is essential in RSV inducing apoptosis in NKTCL cell lines, an ATM inhibitor KU55933 was used. KU55933 diminished the expression level of pATM, pChk2 and pp53 activated by RSV, and reversed RSV-induced cell apoptosis. Furthermore, we found that RSV had synergistic effect on inhibiting NKTCL and activating DDR pathway in combination with ionizing radiation or etoposide, a chemotherapy medication used for the treatments of a number of types of cancer by causing DNA strands to break and promoting apoptosis. These results suggest that DDR pathway plays a crucial role in RSV inducing NKTCL cell apoptosis.

Several viruses have been shown to interact with and/or affect components of the DNA damage pathway [24, 56, 57]. Researchers have demonstrated that during the EBV lytic cycle, DDR pathway is activated in response to large amounts of exogenous double stranded DNA products. During this process, Zta induces phosphorylation of ATM, Chk2, and p53 in a DNA binding dependent manner [10, 58, 59]. Since NKTCL is highly associated with EBV, we examined wheather RSV had any influence on EBV lytic cycle. As shown in our results, RSV induced the expression of EBV lytic gene Zta, without influencing on the latent gene LMP1. So we proposed that the activation of DDR by RSV in NKTCL cell lines may occur partly through inducing the expression of lytic gene Zta, althouth LMP1 is thought to be important in the pathogenesis of NKTCL [7]. However, the molecular mechanism underlying how RSV increases Zta level is obscure.

**Fig. 7:**
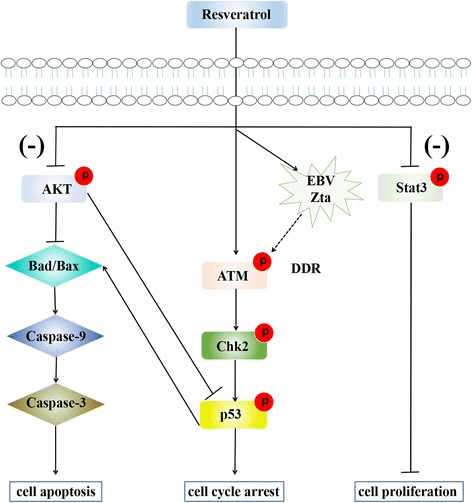
A model of RSV inhibiting NKTCL cells. We make a hypothesis about the mechanisms of RSV inhibiting NKTCL. RSV inhibits NKTCL cell proliferation, induces cell cycle arrest and apoptosis through down-regulating the activities of AKT and Stat3 and inducing DDR. Down-regulated pAKT leads to up-regulation of pro-apoptotic protein Bad and Bax, which then activate the cleaved caspase-9 and caspase-3, resulting in cell apoptosis via mitochondrial pathway. On the other hand, down-regulated AKT causes p53 activated. By inducing DDR directly or via increasing Zta of EBV, RSV phosphorylates ATM, Chk2 and p53, subsequently inhibits Cyclin A2, which leads to S phase cell cycle arrest. p53 can also up-regulate Bax and Bad and activate caspase cascade, which ultimately leads to apoptosis

Taken together, we can make a hypothesis: RSV inhibits NKTCL cell proliferation, induces cell cycle arrest and apoptosis through down-regulating the activities of AKT and Stat3 and inducing DDR. Down-regulated AKT leads to up-regulation of pro-apoptotic protein Bax and Bad, which then activate the cleaved caspase-9 and caspase-3, resulting in cell apoptosis via mitochondrial pathway. On the other hand, down-regulated AKT causes p53 activated. By inducing DDR directly or via increasing Zta of EBV, RSV phosphorylates ATM, Chk2 and p53, subsequently inhibits Cyclin A2, which leads to S phase cell cycle arrest. p53 can also up-regulate Bax and Bad and activate caspase cascade, which ultimately leads to apoptosis (Fig. 7).

## Conclusion

In conclusion, our results provide in vitro evidence that RSV produces anti-tumor effect by activating DDR pathway in an ATM/Chk2/p53 dependent manner. RSV may be worthy for further study as an anti-tumor drug for NKTCL treatment.

## Additional files

Additional file 1: Figure S1. Stat3 phosphorylation is directly associated with cell proliferation in NKTCL cells. A. SNT-8 and SNK-10 cells were exposed to AG490 in the concentration of 0 μM (Control), 25 μM, 50 μM, 100 μM, or 150 μM. The cell proliferation viability was measured using CCK-8 assay at 24, 48 and 72 h after treatment. B. SNT-8 and SNK-10 cells were exposed to AG490 (50 μM) for 12, 24 or 36 h. The phosphorylation level of JAK2 and Stat3 was analyzed by western blot analysis. (TIFF 4114 kb)

Additional file 2: Figure S2. RSV in combination with etoposide (ETO) has synergistic effect on cell proliferation and DDR in NKTCL cells. A. SNT-8 and SNK-10 cells were incubated with RSV (25 μM), etoposide (ETO, 1 μM), or RSV in combination with etoposide (RSV + ETO). After 24 and 48 h, cell viability was determined using CCK-8 assay. Each value represents the mean ± SD of 3 independent experiments. B. SNT-8 and SNK-10 Cells were incubated with RSV (25 μM), etoposide (ETO, 1 μM), or RSV in combination with etoposide (RSV + ETO) for 6 h. The protein levels of pATM (S1981), ATM, γH2A.X (S139), pChk2 (T68), pp53 (S15), p53 were monitored. *p < 0.05 RSV + ETO vs RSV, ^#^ *p* < 0.05 RSV + ETO vs ETO. (TIFF 3788 kb)

Additional file 3: Figure S3. RSV in combination with ionizing radiation (IR) has synergistic effect on cell apoptosis and DDR in NKTCL cells. SNT-8 and SNK-10 cells were exposed to RSV (25 μM), IR (4 Gy), or RSV in combination with IR (RSV + IR) for 24 h. A. Cell apoptosis was analyzed by Flow cytometry in each group. B. The protein levels of pATM (S1981), ATM, γH2A.X (S139), pChk2 (T68), pp53 (S15), p53 were measured. (TIFF 1959 kb)

## Abbreviations

ATM: Ataxia telangiectasia mutated kinase
Chk2: Checkpoint kinase 2
DDR: DNA damage response
DSBs: DNA double-strand breaks
EBV: Epstein-Barr virus
NKTCL: Extranodal NK/T cell lymphoma
RSV: Resveratrol
