# Supplementary figures and images for: Resveratrol inhibits Extranodal NK/T cell lymphoma through activation of DNA damage response pathway
Source: J Exp Clin Cancer Res. 2017 Sep 26;36:133. doi: 10.1186/s13046-017-0601-6 (PMC5615630; doi:10.1186/s13046-017-0601-6)

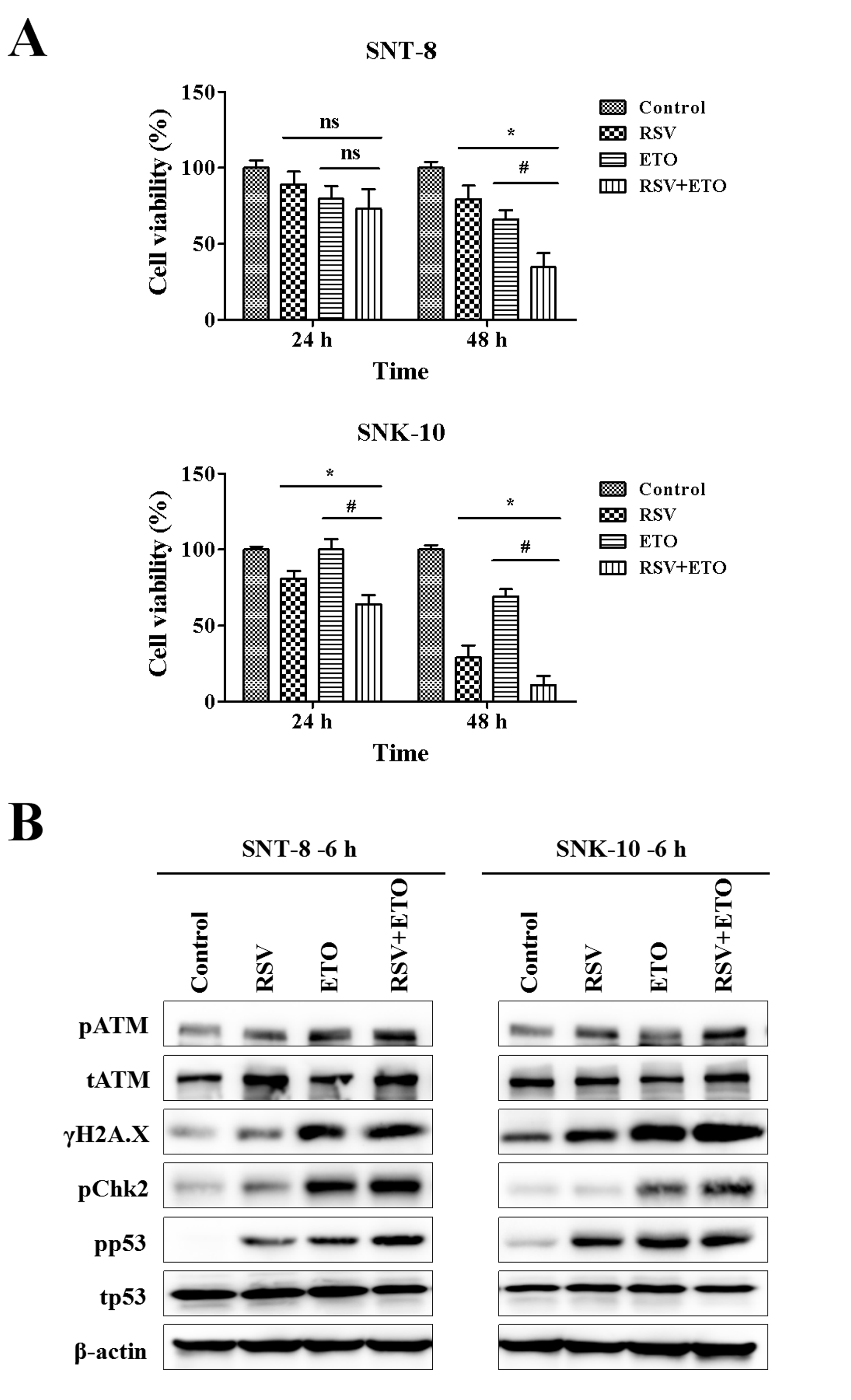

Supplement: Supplementary file 2 — RSV in combination with etoposide (ETO) has synergistic effect on cell proliferation and DDR in NKTCL cells. A. SNT-8 and SNK-10 cells were incubated with RSV (25 μM), etoposide (ETO, 1 μM), or RSV in combination with etoposide (RSV + ETO). After 24 and 48 h, cell viability was determined using CCK-8 assay. Each value represents the mean ± SD of 3 independent experiments. B. SNT-8 and SNK-10 Cells were incubated with RSV (25 μM), etoposide (ETO, 1 μM), or RSV in combination with etoposide (RSV + ETO) for 6 h. The protein levels of pATM (S1981), ATM, γH2A.X (S139), pChk2 (T68), pp53 (S15), p53 were monitored. *p < 0.05 RSV + ETO vs RSV, # p < 0.05 RSV + ETO vs ETO. (TIFF 3788 kb) [file 13046_2017_601_MOESM2_ESM.tif]

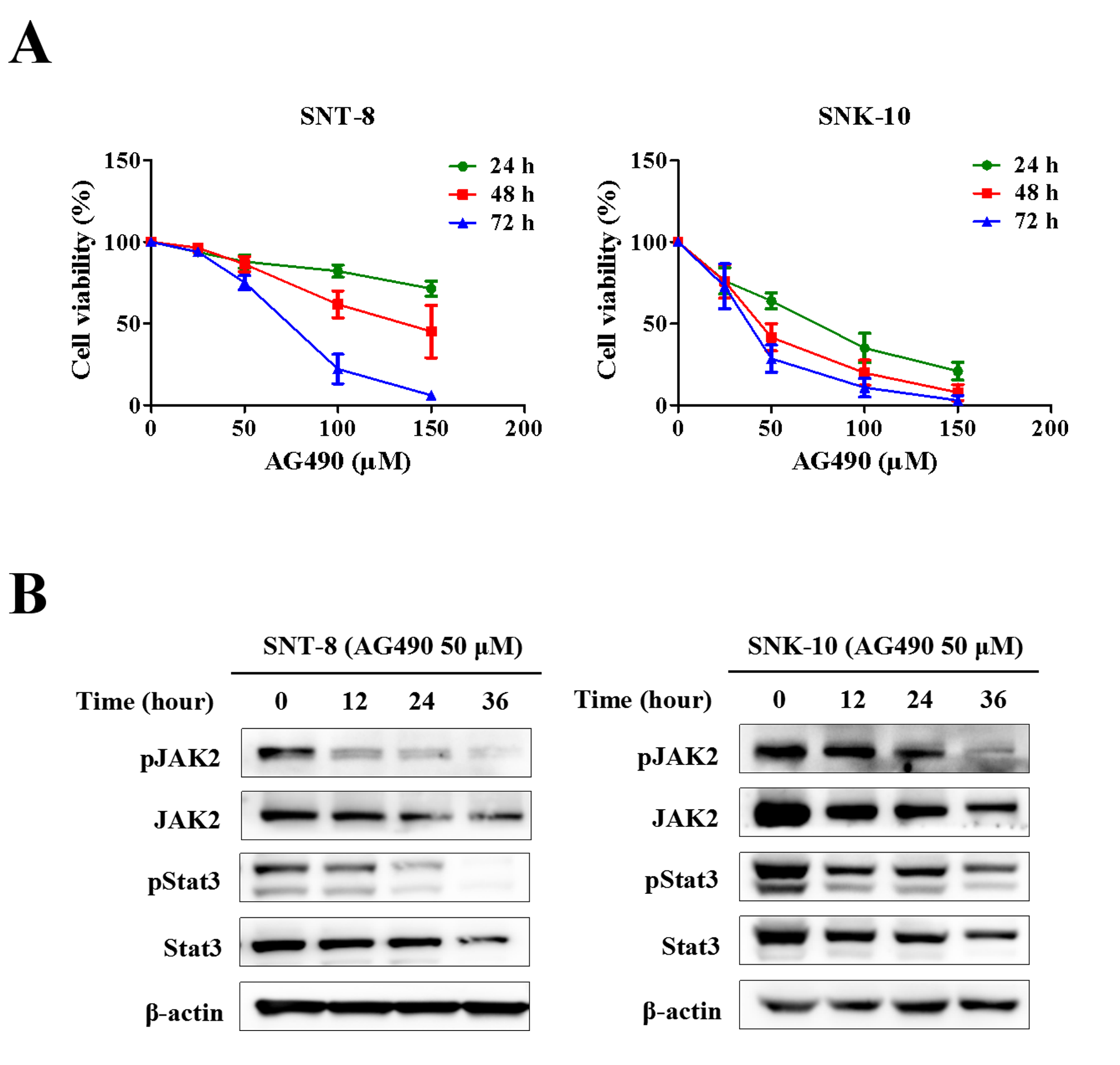

Supplement: Supplementary file 3 — RSV in combination with ionizing radiation (IR) has synergistic effect on cell apoptosis and DDR in NKTCL cells. SNT-8 and SNK-10 cells were exposed to RSV (25 μM), IR (4 Gy), or RSV in combination with IR (RSV + IR) for 24 h. A. Cell apoptosis was analyzed by Flow cytometry in each group. B. The protein levels of pATM (S1981), ATM, γH2A.X (S139), pChk2 (T68), pp53 (S15), p53 were measured. (TIFF 1959 kb) [file 13046_2017_601_MOESM1_ESM.tif]
